# Did the pandemic have an impact on influenza vaccination attitude? a survey among health care workers

**DOI:** 10.1186/1471-2334-11-87

**Published:** 2011-04-07

**Authors:** Bilgin Arda, Raika Durusoy, Tansu Yamazhan, Oğuz Reşat Sipahi, Meltem Taşbakan, Hüsnü Pullukçu, Esra Erdem, Sercan Ulusoy

**Affiliations:** 1 Department of Infectious Diseases and Clinical Microbiology, Ege University Medical School, Izmir, Turkey; 2 Department of Public Health, Ege University Medical School, Izmir, Turkey

## Abstract

**Background:**

Health care workers' (HCWs) influenza vaccination attitude is known to be negative. The H1N1 epidemic had started in mid 2009 and made a peak in October-November in Turkey. A national vaccination campaign began on November 2^nd^, 2009. Despite the diligent efforts of the Ministry of Health and NGOs, the attitudes of the media and politicians were mostly negative. The aim of this study was to evaluate whether HCWs' vaccination attitudes improved during the pandemic and to assess the related factors.

**Methods:**

This cross-sectional survey was carried out at the largest university hospital of the Aegean Region-Turkey. A self-administered questionnaire with 12 structured questions was applied to 807 HCWs (sample coverage 91.3%) before the onset of the vaccination programme. Their final vaccination status was tracked one week afterwards, using immunization records. Factors influencing vaccination rates were analyzed using ANOVA, t-test, chi-square test and logistic regression.

**Results:**

Among 807 participants, 363 (45.3%) were doctors and 293 (36.6%) nurses. A total of 153 (19.0%) had been vaccinated against seasonal influenza in the 2008-2009 season. Regarding H1N1 vaccination, 143 (17.7%) were willing to be vaccinated vs. 357 (44.2%) unwilling. The number of indecisive HCWs was 307 (38.0%) one week prior to vaccination. Only 53 (11.1%) stated that they would vaccinate their children. Possible side effects (78%, n = 519) and lack of comprehensive field evaluation before marketing (77%, n = 508) were the most common reasons underlying unwillingness or hesitation.

Among the 749 staff whose vaccination status could be tracked, 228 (30.4%) actually received the H1N1 vaccine. Some of the 'decided' staff members had changed their mind one week later. Only 82 (60%) of those willing, 108 (37%) of those indecisive and 38 (12%) of those unwilling were vaccinated.

Indecisive HCWs were significantly younger (p = 0.017). Females, nurses, and HCWs working in surgical departments were more likely to reject vaccination (p < 0.05). Doctors, HCWs working in medical departments, and HCWs previously vaccinated against seasonal influenza were more likely to accept vaccination (p < 0.05). Being younger than 50 and having been vaccinated in the previous season were important predictors of attitude towards pandemic influenza vaccination.

**Conclusions:**

Vaccination rates increased substantially in comparison to the previous influenza season. However, vaccination rates could have been even higher since hesitation to be vaccinated increased dramatically within one week (only 60% of those willing and the minority of those indecisive were finally vaccinated). We speculate that this may be connected with negative media at the time.

## Background

Pandemic H1N1 influenza had become a major public health problem in 2009. Uncertainties about the disease's severity and its mortality had caused wide panic and worry, but anxiety declined as awful disaster scenarios did not come true [1]. The social impact of the pandemic was also concerning [2].

Health care workers (HCWs) in hospitals can be a source of infection for diseases that spread quickly like influenza. Thus the vaccination of this group is considered as an important strategy in the prevention of seasonal or pandemic influenza. The under-vaccination of health care workers to seasonal influenza is a well studied topic [3]. In order to manage HCW services better in a future pandemic, it would be interesting to find out whether their attitudes change during a pandemic and whether the same determinants operate or there are new predictors or different reasons for not receiving the vaccine [4,5].

The population of Turkey is 71.5 million. For the 2009-2010 season, the Turkish Ministry of Health had made arrangements for the supply of about 43 million doses of the pandemic H1N1 vaccine [6]. The implementation of the vaccine started on November 2^nd^, 2009. However, debates on vaccine effectiveness, safety and side effects were going on like in the rest of the world. Despite efforts of the academics and National Medical Associations, the propaganda carried out by the media and the Prime Minister about vaccination and pandemic was mostly negative [7]. The aims of this study were:

- To evaluate the intention on vaccination against pandemic H1N1 and final vaccination status of HCWs

- To compare their newly valid vaccination rates with their vaccination rates in the previous season,

- To assess factors influencing vaccine uptake,

- To determine reasons for vaccine declination,

- To discuss political and communication issues that might have operated at the community-level and influenced HCWs during the pandemic.

## Methods

### Setting

Ege University Medical School's hospital is the largest tertiary-care educational university hospital in the Aegean Region. It is a 2000-bed facility located in Izmir which has more than 3 500 000 inhabitants. The hospital is part of a large medical educational and research institution with approximately 1200 medical students and 2079 HCWs. HCWs were the target group of the study. A sample size of 884 was calculated with prevalence 50% for unknown vaccination rate, d = 0.025 and 95% confidence level.

Seasonal influenza vaccination has been administered free of charge to HCWs in Turkey since 2006. In 2009-2010 season, vaccination against pandemic H1N1 and seasonal influenza started simultaneously on November 2^nd ^2009.

Before the study, approval was obtained from the hospital's managers to conduct this study. Since it was not an experimental or interventional study, ethical committee approval was not required.

In our setting, HCWs' vaccinations are organized by the hospital infection control committee and applied in every department of the hospital. On December 1^st^, official vaccination lists were obtained from the hospital management and actual vaccination statuses were matched with questionnaires using the names provided on questionnaires. Particular attention was paid to the confidentiality of the data. Analyses were done and presented anonymously.

### Questionnaire

The study tool was a self-administered questionnaire. It was prepared by infectious diseases & clinical microbiology and public health specialists for this cross-sectional survey. It included seven questions on socio-demographic variables and job history, two questions on past seasonal influenza vaccination status, two questions on attitudes towards vaccinating themselves and their children, and one question on the reason(s) for not being vaccinated in the case of hesitation or unwillingness.

The questionnaire was administered between October 26 and 30^th^, 2009, just before the beginning of vaccination campaign. HCWs were visited by the study team in their departments. Due to time restrictions to collect data before the start of vaccination, convenience sampling was applied in the departments and in the refectory. A total of 810 HCWs could be contacted and only three of them refused to participate. Thus, 91.3% (n = 807) of the intended sample size of 884 was reached. A non-responder analysis was conducted. There was no significant difference in the distribution of profession and departments among responders and non-responders (p = 0.249 and 0.123, respectively).

### Statistical analysis

Univariate analyses were conducted to explore the impact of gender, age, presence of children, duration of work in years, profession, department, influenza risk perception, previous vaccination against seasonal influenza and perceived influenza risk on HCWs' attitudes towards vaccinating themselves (willing/unwilling/indecisive), risk perception and their actual vaccination status (yes/no) using ANOVA, t-test (age, duration of work in years) and chi-square tests. A multivariate stepwise logistic regression (selection method 'Enter') was performed with the variables that were found significant in univariate analysis with the exception of ever being vaccinated against seasonal influenza in the past five years which was correlated with vaccination in the previous season. The difference between the proportions of vaccination against seasonal influenza in 2008-2009 and pandemic influenza in 2009-2010 was compared with McNemar test.

The level of statistical significance was p < 0.05. Analyses were performed with SPSS 15.0. We achieved 97% power to detect an effect size of 0.1624 using a 3-degrees-of-freedom chi-square test with alpha 0.05 when analyzing the effect of profession on vaccination status.

## Results

A total of 807 HCWs were interviewed before the first day of the vaccination programme. The departments with highest numbers of participants were internal medicine (19.1%, n = 151), general surgery (9.3%, n = 74) and anaesthesia & reanimation (5.2%, n = 41).

Among participants, 363 were doctors (45.3%), 293 were nurses (36.6%), followed by 111 health technicians (13.9%) and 34 others (4.2%), with a female predominance (66.5%, n = 536). Mean age of participants was 35.1 ± 8.8 (range 19 - 66, median 34). Mean duration of work in years was 12.1 ± 9.0 (range 1 - 44, median 10).

Regarding pandemic H1N1 vaccination attitude, only 143 (17.7%) were willing to get vaccinated. Among HCWs, 496 (60.6%) had children aged 6 months to 24 years and only 53 (11.1%) were willing to vaccinate them. Among participants, 634 (78.9%) perceived themselves under risk of influenza, 153 (19.0%) were vaccinated against seasonal influenza in the 2008-2009 season and 305 (37.9%) were in the last five years. The responses of HCWs to these questions are shown in detail in Table 1.

**Table 1:** HCW's risk perception, previous seasonal influenza vaccination practices and attitudes toward vaccination against H1N1

	n	%
Risk perception of being among the risk groups of pandemic H1N1		
Perceives the risk	634	78.9
Does not perceive the risk	170	21.1
Vaccination against seasonal influenza at least once in the past five years		
Vaccinated	305	37.9
Not vaccinated	490	60.9
Don't remember	10	1.2
Vaccination against seasonal influenza in the last season		
Vaccinated	153	19.0
Not vaccinated	643	79.8
Don't remember	10	1.2
Intention to be vaccinated against H1N1		
Will be vaccinated	143	17.7
Will not be vaccinated	357	44.2
Indecisive	307	38.0
Intention to vaccinate their children 6 months to 24 years old against H1N1		
Will vaccinate them	53	11.1
Will not vaccinate them	241	50.3
Indecisive	182	38.6

The most common underlying reason for unwillingness or hesitation concerning pandemic H1N1 vaccination was possible side effects, followed by the lack of comprehensive field evaluation before marketing. The distribution and variety of reasons underlying hesitation and unwillingness are shown in Table 2. Indecisive participants were more likely to be concerned about possible side effects while participants rejecting the vaccine more likely thought that the disease was mild or that contracting the disease was safer (p < 0.05).

**Table 2:** Reasons underlying unwillingness or hesitation to be vaccinated against pandemic H1N1 (n, %)

Reasons of unwillingness or hesitation	Will not be vaccinated	Indecisive	Total
Possible side effects	267 (74.8)	251 (82.0)	518 (78.1)
Lack of comprehensive field evaluation before marketing	266 (74.5)	241 (78.8)	507 (76.5)
Implementation in our country before wide implementation in other countries	199 (55.9)	184 (60.1)	383 (57.9)
Vaccines which will be administered here are different than the vaccines in the US	153 (42.9)	137 (44.9)	290 (43.8)
Do not have trust in the effectiveness of the vaccine	157 (44.0)	114 (37.3)	271 (40.9)
Do not trust Ministry of Health's practices	147 (41.2)	104 (34.0)	251 (37.9)
Due to the risk of acquiring H1N1 following vaccination	71 (19.9)	66 (21.6)	137 (20.7)
The disease is mild	59 (16.5)	34 (11.1)	93 (14.0)
Contracting the disease is safer	34 (9.5)	13 (4.2)	47 (7.1)
Other	30 (8.4)	15 (4.9)	45 (6.8)

Mean age of indecisive HCWs was significantly lower than participants rejecting the vaccine. Females, nurses, and HCWs working in the surgical departments were more likely to reject vaccination (p < 0.05). To explore possible effect modification by profession, a stratified analysis was done. Among female participants, only 20 (7.0%) of nurses were willing to be vaccinated vs. 51 (20.6%) among females with other professions (χ^2 ^= 21.211, p < 0.001). Doctors, HCWs working in internal departments and HCWs that were previously vaccinated against seasonal influenza were more likely to accept vaccination (p < 0.05). Health technicians and other professions did not have a significantly different attitude than others (Table 3). A similar pattern was observed for attitudes toward vaccinating their children, except for the disappearing effect of gender.

**Table 3:** Factors influencing attitudes toward getting vaccinated against pandemic influenza

	Intention to get vaccinated			
**Factor**	**Yes**	**No**	**Indecisive**	**p**

Mean age, yr	35.8 ± 8.9	35.7 ± 8.8^a^	33.9 ± 8.6^a^	0.016
Mean duration of work, yr	12.5 ± 9.4	12.6 ± 9.0	11.1 ± 8.9	0.084
Gender (n, %)				
Female	72 (13.4)	246 (45.9)	218 (40.7)	<0.001
Male	71 (26.3)	110 (40.7)	89 (33.0)	
Having children				
Yes	73 (15.2)	238 (49.7)	168 (35.1)	0.001
No	67 (21.5)	113 (36.3)	131 (42.1)	
Profession^b^				
Doctor	94 (25.9)	139 (38.3)	130 (35.8)	<0.001
Nurse	21 (7.2)	144 (49.1)	128 (43.7)	<0.001
Health technician	16 (14.4)	55 (49.5)	40 (36.0)	0.407
Other	10 (29.4)	15 (44.1)	9 (26.5)	0.128
Department^b^				
Internal medical departments	89 (21.8)	153 (37.5)	166 (40.7)	<0.001
Surgical departments	31 (10.9)	148 (52.1)	105 (37.0)	<0.001
Basic medical sciences	15 (21.1)	30 (42.3)	26 (36.6)	0.742
Other units	6 (20.7)	14 (48.3)	9 (31.0)	0.689
Perceived risk of swine flu				
Yes	131 (20.7)	234 (36.9)	269 (42.4)	<0.001
No	12 (7.1)	121 (71.2)	37 (21.8)	
Ever vaccinated against seasonal influenza in the past 5 years				
Yes	76 (24.9)	111 (36.4)	118 (38.7)	<0.001
No	65 (13.1)	241 (49.3)	184 (37.6)	
Vaccinated against seasonal influenza last year				
Yes	49 (31.8)	48 (31.2)	57 (37.0)	<0.001
No	92 (14.3)	303 (47.2)	247 (38.5)	

^a ^Groups creating the difference according to Bonferroni's Post Hoc test

^b ^Each category compared with the total of other categories

Among individual departments with >10 participants, the three clinics with highest intent for vaccination were respiratory diseases (42.9%, n = 15 of 35), infectious diseases (40.0%, 6 of 15) and cardiovascular surgery (35.7%, 5 of 14), respectively. The highest rates of opposition were observed in plastic and reconstructive surgery (75.0%, n = 12 of 16), otorhinolaringology (64.3%, 9 of 14) and general surgery (60.8%, 45 of 74) clinics.

Pandemic H1N1 vaccines were administered to volunteering health HCWs during the week following the interviews, along with the simultaneous administration of seasonal influenza vaccine. Vaccination status could be tracked among 92.8% (n = 749) of participants, who had provided their full names on the questionnaires. Of these, 228 (30.4%) were vaccinated against H1N1 and 521 (69.6%) were not. The distribution of their actual vaccination status according to their intention in the previous week and their seasonal influenza vaccination status for the previous year is presented in Table 4.

**Table 4:** Actual vaccination status of HCWs according to their intention and past seasonal influenza vaccination (n, %)

Intention and previous vaccination	Vaccinated against pandemic H1N1
Will be vaccinated against H1N1^a^	
Yes	82 (59.9)
No	38 (11.8)
Indecisive	108 (37.1)
	
Seasonal influenza vaccination in the past year^b^	
Yes	62 (43.7)
No	163 (27.3)
	
Total	228 (30.4)

^a ^χ^2 ^= 114.557, p < 0.001

^b ^McNemar test, p < 0.001

When risk perception was analyzed according to age categories, a significant difference was noted. The perceived risk decreased with age: 81.8%, 82.5%, 75.7% and 59.3% for the age categories <30, 30-39, 40-49 and ≥50, respectively (χ^2 ^= 18.270, p < 0.001, n = 225, 222, 137, 35, respectively). Such a trend was not observed for actual vaccination rates, but vaccination rate was significantly lower for HCWs ≥50 years old. The effects of possible factors that could influence vaccination status are shown in Table 5 along with factors that could have an impact on risk perception.

**Table 5:** Factors influencing perceived risk of influenza and final H1N1 vaccination status

	Perceived risk			Vaccination status		
**Factor**	**Yes**	**No**	**OR (95% CI)**	**Vaccinated**	**Not vaccinated**	**OR (95% CI)**

Age						
<50 years	584 (80.6)	141 (19.4)	2.84 (1.64-4.93)^a^	214 (31.7)	462 (68.3)	2.13 (1.06-4.30)^a^
50+	35 (59.3)	24 (40.7)	1	10 (17.9)	46 (82.1)	1
Mean duration of work, yr						
<5 years	182 (80.9)	43 (19.1)	1.50 (0.98-2.28)	74 (33.8)	145 (66.2)	1.15 (0.79-1.68)
5-9	110 (83.3)	22 (16.7)	1.77 (1.04-2.99)^a^	43 (34.7)	81 (65.3)	1.20 (0.76-1.88)
10-14	96 (82.1)	21 (17.9)	1.62 (0.94-2.77)	21 (20.4)	82 (79.6)	0.58 (0.34-1.00)
15+	215 (73.9)	76 (26.1)	1	83 (30.7)	187 (69.3)	1
Gender (n, %)						
Male	201 (74.4)	69 (25.6)	1	82 (32.3)	172 (67.7)	1
Female	433 (81.2)	100 (18.8)	1.49 (1.05-2.11)^a^	146 (29.6)	348 (70.4)	0.88 (0.64-1.22)
Having children						
No	259 (83.5)	51 (16.5)	1	100 (34.1)	193 (65.9)	1
Yes	361 (75.7)	116 (24.3)	0.61 (0.43-0.88)^a^	124 (28.2)	315 (71.8)	0.76 (0.55-1.05)
Profession						
Doctor	264 (72.9)	98 (27.1)	1	131 (38.5)	209 (61.5)	1
Nurse	253 (86.9)	38 (13.1)	2.47 (1.64-3.73)^a^	65 (24.5)	200 (75.5)	0.52 (0.36-0.74)^a^
Health technician	86 (77.5)	25 (22.5)	1.28 (0.77-2.11)	25 (23.1)	83 (76.9)	0.48 (0.29-0.79)^a^
Other	26 (76.5)	8 (23.5)	1.21 (0.53-2.76)	6 (18.8)	26 (81.3)	0.37 (0.15-0.92)^a^
Department						
Internal medical departments	333 (81.8)	74 (18.2)	1	139 (35.5)	252 (64.5)	1
Surgical departments	220 (78.0)	62 (22.0)	0.79 (0.54-1.15)	50 (19.1)	212 (80.9)	0.43 (0.30-0.62)^a^
Basic medical sciences	49 (69.0)	22 (31.0)	0.50 (0.28-0.87)^a^	27 (41.5)	38 (58.5)	1.29 (0.75-2.20)
Other units	22 (75.9)	7 (24.1)	0.70 (0.29-1.70)	11 (39.3)	17 (60.7)	1.17 (0.53-2.58)
Perceived risk of pandemic influenza						
Does not perceive risk	-	-	-	33 (21.7)	119 (78.3)	1
Perceives the risk	-	-	-	195 (32.8)	399 (67.2)	1.76 (1.16-2.69)^a^
Ever vaccinated against seasonal influenza in the past 5 years						
No	359 (73.6)	129 (26.4)	1	121 (26.6)	334 (73.4)	1
Yes	266 (87.5)	38 (12.5)	2.52 (1.70-3.73)^a^	106 (37.2)	179 (62.8)	1.64 (1.19-2.25)^a^
Vaccinated against seasonal influenza last year						
No	491 (76.8)	148 (23.2)	1	163 (27.3)	434 (72.7)	1
Yes	135 (87.7)	19 (12.3)	2.14 (1.28-3.58)^a^	62 (43.7)	80 (56.3)	2.06 (1.42-3.01)^a^

^a ^p < 0.05

Though HCWs who perceived themselves under risk of pandemic H1N1 were significantly younger (mean ages 34.4 ± 8.3 and 37.5 ± 9.9, respectively, t = -3.754, p = < 0.001), there was no significant difference in mean ages of vaccinated and unvaccinated HCWs (34.5 ± 8.2 and 35.2 ± 9.1 years, respectively, t = -0.941, p = 0.347). Likewise, HCWs who felt under risk had a shorter duration of employment (11.5 ± 8.7 vs. 14.0 ± 10.1 years, t = -2.831, p = 0.005) while there was no significant difference in mean duration of employment among vaccinated and unvaccinated HCWs (11.2 ± 8.6 vs. 12.3 ± 9.3 years, t = -0.1486, p = 0.138). There was no significant difference in the past seasonal influenza vaccination rates among different professions and departments (chi-square p > 0.05).

Among departments with >10 participants, actual vaccination rates were the highest in infectious diseases (76.9%, n = 10 of 13), respiratory diseases (70.6%, 24 of 34) and campus outpatient clinics (57.1%, 8 of 14) and the lowest in otorhinolaringology (0.0%, 0 of 14), plastic and reconstructive surgery (0.0%, 0 of 13), general surgery clinics (4.5%, 3 of 66).

According to the multivariate logistic regression analysis including the variables in Table 6, HCWs aged <50 years, HCWs perceiving a higher risk of pandemic H1N1 and HCWs vaccinated against seasonal influenza in the 2008-09 season were significantly more likely to get vaccinated. However, not being a doctor but a nurse or a health technician and working in a surgical department were significant variables associated with non-vaccination. Odds ratios and 95% confidence intervals are presented in Table 6.

**Table 6:** Multivariate logistic regression analysis on determinants of H1N1 vaccination status

Factor	OR (95% CI)
Age <50 years	2.82 (1.28-6.19)^a^
Female gender	0.82 (0.54-1.23)
Profession (ref. = doctor)	
Nurse	0.61 (0.39-0.95)^a^
Health technician	0.43 (0.25-0.74)^a^
Other	0.24 (0.08-0.77)^a^
Department (ref. = internal departments)	
Surgical departments	0.45 (0.30-0.67)^a^
Basic medical sciences	1.51 (0.83-2.76)
Other units	1.62 (0.63-4.20)
Perceived risk of swine flu	1.61 (1.02-2.54)^a^
Vaccinated against seasonal influenza last year	2.19 (1.46-3.29)^a^

^a ^p < 0.05

## Discussion

HCW had considerable hesitation to be vaccinated with the pandemic H1N1 vaccine despite a higher degree of preparedness (or at least indecisiveness) to be vaccinated shortly before the vaccination programme. Only 60% of those willing and a minority of those indecisive were finally vaccinated. Factors that have likely to do with the specific H1N1 vaccine, such as possible side effects and lack of comprehensive field evaluation were the most frequent reasons given for not being vaccinated.

Although the Advisory Committee on Immunization Practices (ACIP) had announced that HCWs should be a high priority group for vaccination efforts, their influenza vaccination rates remained low worldwide both for seasonal and pandemic influenza [8-10]. Seasonal vaccination estimates of U.S. HCWs at 62% were higher than their usual seasonal vaccination rates. However, the estimated percentage of HCWs who received full vaccine coverage for both pandemic and seasonal influenza was only 35% during the 2009-2010 season, similar to previous seasonal vaccination rates [8]. Intention for the pandemic vaccination rate that was 22% in a study from Greece was lower than ours [9]. As an exception, a high rate was reached in the Netherlands. They applied a vaccination campaign towards general practitioners with an increase in seasonal vaccination rate from 36% to 63% and a pandemic vaccination rate of 85% [11].

In our study the most important reason of unwillingness or hesitation against H1N1 vaccine was vaccine safety issues. The same reason was also the most prominent in the U.S. and Greece [8,9]. We think that several additional factors might have contributed to the highly negative aspects about pandemic influenza vaccination:

i) Discussion shows comprised most of the media channels' news content both in October and November 2009. Some of the health, academic, media and governmental authorities were against vaccination. The announcement by the Prime Minister of Turkey, Recep Tayyip Erdogan, on November 3^rd^, 2009 was that he personally would not get vaccinated. Contrary to him the Turkish Minister of Health Recep Akdag was working hard to promote vaccination. This conflict might have led to further confusion and disinformation, especially among the public and also among the health staff.

ii) Izmir is the most important and populated city among the 13 cities that did not give majority support to the political party in power. Additionally, the continuous struggles between the universities and the government might have also amplified the negative propaganda [12,13]. The fact that 34% of the indecisive and 41% of the unwilling staff did not trust Ministry of Health's practices supports this possibility.

iii) The propaganda about the possible unknown side effects (especially neurologic side effects and triggering of autoimmunity) of the adjuvants.

iv) The fact that our setting is a very big hospital might also have caused a low vaccination rate as suggested by an earlier study from Greece [14].

In a study about the correlation between the sources of information and acceptability of the pandemic vaccine, it was found that the scientific literature was supporting the administration more strongly than Google and the press releases of politicians had a negative impact on the vaccination campaign. It was also reported that the propaganda of the media is among the important reasons underlying the failure of the vaccination campaign [15].

In our study, perception of being at risk for influenza was another factor that was strongly associated with both intention to vaccination and uptake, like in other studies [8,16,17]. Responders who thought that their patient population was under high risk of influenza were more likely to be vaccinated and to agree with statements regarding influenza disease and influenza vaccination of HCW [18]. This might explain the higher rates of vaccination we found in respiratory diseases and infectious diseases & clinical microbiology departments where most of the H1N1 flu patients are hospitalized.

In the present study, it was found that i) indecisive HCWs were younger, ii) doctors were more willing to be vaccinated while nurses were less willing, iii) HCWs in internal medical departments were more willing and staff in surgical departments less willing to be vaccinated. The effect of age and gender that was found significant for intention disappeared for actual vaccination status, while trends among professions and departments continued. Doctors had a higher vaccination rate in other studies as well [9,19]. Within the population of HCWs, nurses have been shown to have lower influenza vaccination rates than physicians [18,20]. In a previous study this was partly explained by their level of knowledge [18]. It has been suggested that nursing staff are more likely to refuse vaccine because of commonly held misconceptions about adverse effects and efficacy [20].

Surprisingly, basic medical science workers had the highest rate of vaccination while they had significantly lower perceived risk. This might be due to the over-representation of the microbiology department (49.2%) among basic medical science workers which had one of the highest vaccination rates.

The association found between longer duration of employment and/or older age and higher vaccination rate was not observed in our study, probably due to the fact that younger age groups were more vulnerable for the H1N1 pandemic [21,22].

There are several limitations to this study. First, this study was conducted in only one university hospital which might not represent all HCWs in Izmir or Turkey. However, our hospital is the largest university hospital both in Izmir and the Aegean Region of Turkey, it has a high number of HCWs and the study sample comprised more than 800 HCWs. Another limitation is that HCWs' knowledge about pandemic influenza, its vaccine and the presence of a chronic condition targeted for H1N1 vaccine were not questioned.

The negative attitudes to the vaccine were not only observed among HCWs but also in the community. Of the 43 million doses of swine flu vaccine that the Turkish Ministry of Health had made arrangements for the 2009-2010 season, 30 millions were cancelled due to the generally low will for the vaccine.

## Conclusions

Low influenza vaccination rate in the HCWs is a global problem. Studies regarding how to increase vaccination rates suggest that i)free of charge vaccines ii)vaccination in 24 h open vaccination centres iii)mobile vaccination cards iv)administrative emphasis and support, v)education vi)signed declination forms vii)use of media campaigns or non-profit organizations that might push politicians and physicians to take further action viii)mandatory vaccination may be suitable interventions [5,23-25]. In spite of the fact that free of charge vaccines and administrative emphasis and support were already present in our sample, attitudes were considerably negative. Nevertheless, their uptake of the pandemic influenza vaccine was higher than their seasonal influenza vaccination rate in the previous season. Our data suggest that efforts should be increased to encourage pandemic influenza vaccination.

## Competing interests

The authors declare that they have no competing interests.

## Authors' contributions

BA conceived of the study, participated in the design and data collection and drafted the manuscript. RD participated in the design and data collection of the study, analyzed the data and drafted the manuscript. TY participated in the design and data collection of the study and helped to draft the manuscript. ORS participated in the design and data collection of the study and drafted the manuscript, MT collected data and helped to draft the manuscript. HP and EE participated in data collection and data entry. SU critically reviewed the text. All authors have read and approved the final manuscript

## Pre-publication history

The pre-publication history for this paper can be accessed here:

http://www.biomedcentral.com/1471-2334/11/87/prepub
